# A Mechanistic Basis for the Coordinated Regulation of Pharyngeal Morphogenesis in *Caenorhabditis elegans* by LIN-35/Rb and UBC-18–ARI-1

**DOI:** 10.1371/journal.pgen.1000510

**Published:** 2009-06-12

**Authors:** Kumaran Mani, David S. Fay

**Affiliations:** Department of Molecular Biology, College of Agriculture, University of Wyoming, Laramie, Wyoming, United States of America; Stanford University Medical Center, United States of America

## Abstract

Genetic redundancy, whereby two genes carry out seemingly overlapping functions, may in large part be attributable to the intricacy and robustness of genetic networks that control many developmental processes. We have previously described a complex set of genetic interactions underlying foregut development in the nematode *Caenorhabditis elegans*. Specifically, LIN-35/Rb, a tumor suppressor ortholog, in conjunction with UBC-18–ARI-1, a conserved E2/E3 complex, and PHA-1, a novel protein, coordinately regulates an early step of pharyngeal morphogenesis involving cellular re-orientation. Functional redundancy is indicated by the observation that *lin-35; ubc-18* double mutants, as well as certain allelic combinations of *pha-1* with either *lin-35* or *ubc-18*, display defects in pharyngeal development, whereas single mutants do not. Using a combination of genetic and molecular analyses, we show that *sup-35*, a strong recessive suppressor of *pha-1*–associated lethality, also reverts the synthetic lethality of *lin-35; ubc-18*, *lin-35; pha-1*, and *ubc-18 pha-1* double mutants. SUP-35, which contains C2H2-type Zn-finger domains as well as a conserved RMD-like motif, showed a dynamic pattern of subcellular localization during embryogenesis. We find that mutations in *sup-35* specifically suppress hypomorphic alleles of *pha-1* and that SUP-35, acting genetically upstream of SUP-36 and SUP-37, negatively regulates *pha-1* transcription. We further demonstrate that LIN-35, a transcriptional repressor, and UBC-18–ARI-1, a complex involved in ubiquitin-mediated proteolysis, negatively regulate SUP-35 abundance through distinct mechanisms. We also show that HCF-1, a *C. elegans* homolog of host cell factor 1, functionally antagonizes LIN-35 in the regulation of *sup-35*. Our cumulative findings piece together the components of a novel regulatory network that includes LIN-35/Rb, which functions to control organ morphogenesis. Our results also shed light on general mechanisms that may underlie developmental genetic redundancies as well as principles that may govern complex disease traits.

## Author Summary

One of the more puzzling aspects of genetics is that the inactivation of many genes fails to produce strong deleterious effects on the organisms that carry those genes. In some cases, however, the combined inactivation of two or more such genes can lead to the expression of robust abnormal phenotypes. These types of synthetic genetic interactions are thought to reflect the presence of functional overlap or redundancy between the involved genes. The root mechanisms that underlie synthetic interactions are thought to be complex and are in most cases poorly understood. Our work here focuses on one case study where we have uncovered the molecular basis underlying a complex set of genetic redundancies in *C. elegans*. More specifically, we have discovered a novel regulatory network that connects eight genes controlling embryonic foregut development in the nematode *C. elegans*. By solving mechanisms of this nature, our analysis provides a means for understanding more generally the principles that govern genetic redundancies. Our work also provides insight into the bases of complex disease traits, where the combined interactions of multiple genetic factors leads to outcomes that determine health or disease.

## Introduction

Genetic redundancy describes the phenomenon in which the combined inactivation of two distinct genes produces a phenotype that is not observed in either single mutant. One of the current challenges facing geneticists and developmental biologists alike is to understand the underlying bases of genetic redundancy at the molecular level. This may in many cases prove to be a difficult undertaking given the complexity of regulatory networks and the many difficulties associated with establishing clear connections between seemingly disparate genes. Nonetheless, redundancy is an issue of great biological importance, as evidenced in *C. elegans*, where most genes fail to show obvious or highly penetrant phenotypes following inhibition or inactivation [1]–[3].

To date, the most intensively studied case of genetic redundancy in *C. elegans* involves the Synthetic Multivulval (SynMuv) genes (for a review, see [4]. The SynMuv genes can in most cases be divided into two principal non-overlapping groups, termed class A and class B [5]. Inhibition of individual class A or class B genes does not typically alter normal patterns of vulval cell induction in hermaphrodites. In contrast, the combined loss in activity of any class A–class B gene pair leads to the ectopic induction of vulval tissue (the Muv phenotype). In addition, a class C group of SynMuv genes has recently been identified; mutations in class C genes are synthetic with mutations in both class A and class B SynMuv genes [6].

Extensive work has shed considerable light on the role of SynMuv genes in vulval development. Namely, most class A and B genes act within the hypodermis, a multi-nucleate epidermal tissue that lies adjacent to the developing vulval precursor cells (VPCs), where they redundantly inhibit the expression of the EGF-like ligand, LIN-3 [7]. Secreted LIN-3 induces vulval cell development through activation of a conserved EGFR–Ras–Map kinase pathway in the VPCs [8]. Thus, in the absence of both class A and class B SynMuv activity, abnormally high levels of LIN-3, secreted by the hypodermis, leads to the hyperinduction of vulval cell fates.

Based on studies in *C. elegans*, *Drosophila*, and mammals, the large majority of proteins encoded by the class B SynMuv gene family function within a conserved set of structurally related transcriptional repressor complexes that include DRM (Dp, Rb and MuvB) and NuRD (nucleosome remodeling and histone deacetylase; reviewed by [4],[9]. Among the components that are common to these complexes are LIN-35, the sole *C. elegans* Retinoblastoma protein (pRb) family ortholog, and EFL-1, a member of the E2F family of transcription factors [10]–[12]. Similar to its role in other systems, LIN-35 acts in large part to mediate the transcriptional repression of E2F target genes [13]. Nevertheless, the precise means by which class A and B SynMuv genes influence the expression of LIN-3 in the hypodermis is currently unclear. Furthermore, the precise molecular functions of class A genes are presently unknown, although a role in transcription has been proposed [4].

We have previously described a forward genetic screen for identifying mutations that show strong synthetic genetic interactions in conjunction with the loss of *lin-35* [14]. This and other work has led to the identification of a diverse array of redundant functions for LIN-35 including roles in cell cycle control [14],[15], cell fate specification [16], asymmetric cell division [17], larval growth [18],[19], fertility [16],[20], organogenesis [20],[21], and organ function [22]. In addition, LIN-35, along with a number of other class B SynMuv genes, has been shown to function non-redundantly in the control of transgene expression [23], RNAi [24],[25], germline and somatic sex-linked apoptosis [26],[27], ribosome biogenesis [28], and the somatic silencing of germline gene expression [13],[25].

In our current work, we have sought to understand the mechanistic basis for the synthetic genetic interactions observed between *lin-35* and two mutations previously identified by our screen, *ubc-18* and *pha-1* [21],[29]. Both *lin-35; ubc-18* and *lin-35; pha-1* double mutants arrest predominantly as L1 larvae and display severe defects in pharyngeal morphogenesis. Furthermore, *ubc-18 pha-1* double mutants are also synthetically lethal, indicating that the functions of these three genes are interconnected [29]. Notably, the genetic interactions between *pha-1* and *lin-35* or *ubc-18* can be observed only under conditions in which *pha-1* activity is weakly compromised. This is because strong loss-of-function mutations in *pha-1* are themselves lethal, and arrested *pha-1* mutant animals display defects in pharyngeal and body morphogenesis [30].

Through an analysis of the suppressor mutation *sup-35*, we demonstrate that SUP-35 acts as an inhibitor of *pha-1* transcription. Furthermore, we show that LIN-35 and UBC-18 act through distinct mechanisms to negatively regulate SUP-35 expression. Thus, the simultaneous loss of *lin-35* and *ubc-18* leads to increased levels of SUP-35, which in turn trigger a reduction in the levels of PHA-1. These findings provide a straightforward explanation for the observed genetic interactions between these genes and more generally provide further insight into the nature of mechanisms that can underlie genetic redundancies.

## Results

### *sup-35* encodes a Zn-finger protein with homology to RMD family members

As described in the Introduction, *lin-35* mutations are strongly synthetic with hypomorphic mutations that affect the *pha-1* locus, leading to strong pharyngeal morphogenesis defects [29]. In addition, recessive mutations in three genetic loci (*sup-35*, *sup-36*, and *sup-37*) were demonstrated to strongly suppress the embryonic- and larval-lethal phenotype of strong loss-of-function *pha-1* mutants [31]. We have previously shown that mutations in *sup-36* and *sup-37* efficiently suppress the synthetic lethality of *lin-35; pha-1* and *lin-35; ubc-18* double mutants [29]. As described below, these and other related synthetic genotypes were also suppressed by mutations in *sup-35*. Thus, to learn more about the interplay between these various factors and their roles in pharyngeal development, we sought to identify the *sup-35* locus.

Previous mapping data had placed *sup-35* on LGIII, ∼0.1 cM to the left of the *pha-1* locus [31]. To identify the gene encoding *sup-35*, we carried out RNAi feeding of 384 clones corresponding to genes in the region proximal to *pha-1*. Two clones, which target the highly related genes Y48A6C.1 and Y48A6C.3, were identified that strongly suppress the embryonic lethality of *pha-1(e2123ts)* mutants (referred to hereafter as *pha-1(ts)*) at the non-permissive temperature of 25°C (Table 1). These RNAi clones also suppress the less severe L1 larval-arrest phenotype of *pha-1(ts)* mutants at intermediate temperature of 20°C (data not shown). Because Y48A6C.1 and Y48A6C.3 share extensive sequence homology (an 878-bp segment present in both genes is 99% identical), each RNAi construct is expected to inhibit both gene products through off-target effects; no additional off targets for these RNAi constructs are predicted. These results suggest that *sup-35* may be encoded by either Y48A6C.1 or Y48A6C.3. However, an additional RNAi construct that is expected to target Y48A6C.1, but not Y48A6C.3, failed to suppress *pha-1(ts)* mutants at 25°C, suggesting that Y48A6C.3 is the relevant locus (data not shown).

**Table 1:** Suppression of multiple genotypes by *sup-35*.

Genotype	Fertile adults (%)
*pha-1(e2123ts)(16°C)*	96.7 (n = 365)
*pha-1(e2123ts); lin-35(RNAi)(16°C)*	2.4 (n = 362)
*sup-35(e2223) pha-1(e2123ts); lin-35(RNAi)(16°C)*	98.3 (n = 301)
*lin-35; pha-1(e2123ts)(16°C)*	0 (n = 148)
*lin-35; sup-35(e2223) pha-1(e2123ts)(16°C)*	98.5 (n = 289)
*pha-1(e2123ts); ubc-18(RNAi)(16°C)*	8.2 (n = 261)
*sup-35(e2223) pha-1(e2123ts); ubc-18(RNAi)(16°C)*	97.6 (n = 284)
*pha-1(e2123ts); Y48A6C.1(RNAi)(25°C)*	100 (n = 353)
*pha-1(e2123ts); Y48A6C.3(RNAi)(25°C)*	100 (n = 357)
*lin-35; pha-1(fd1)* a	0 (n = 248)
*lin-35; pha-1(fd1); sup-35(RNAi)*	54 (n = 598)
*lin-35; ubc-18* a	0 (n = 137)
*lin-35; ubc-18; sup-35(RNAi)*	63 (n = 180)
*ari-1(tm2549); pha-1(e2123ts)(16°C)* b	1.5 (n = 338)
*ari-1(tm2549); pha-1(e2123ts); sup-35(RNAi)(16°C)*	100 (n = 465)
*lin-35; pha-1(RNAi)*	0 (n = 394)
*lin-35; sup-35(tm1810); pha-1(RNAi)*	100 (n = 381)
*lin-35; sup-35(tm1810) ubc-18*	100 (n = 255)

All experiments performed at 20°C unless indicated. Experiments involving *lin-35* and *ubc-18* were performed using the alleles *n745* and *ku354*, respectively. We note that pharyngeal morphology and function was normal in the suppressed fertile adults.

a Double mutants were derived from a parental strain carrying the *lin-35*-rescuing extrachromosomal array, *kuEx119*.

b Double mutants were maintained by propagating low-frequency escapers of the synthetic lethality.

**Figure 1:**
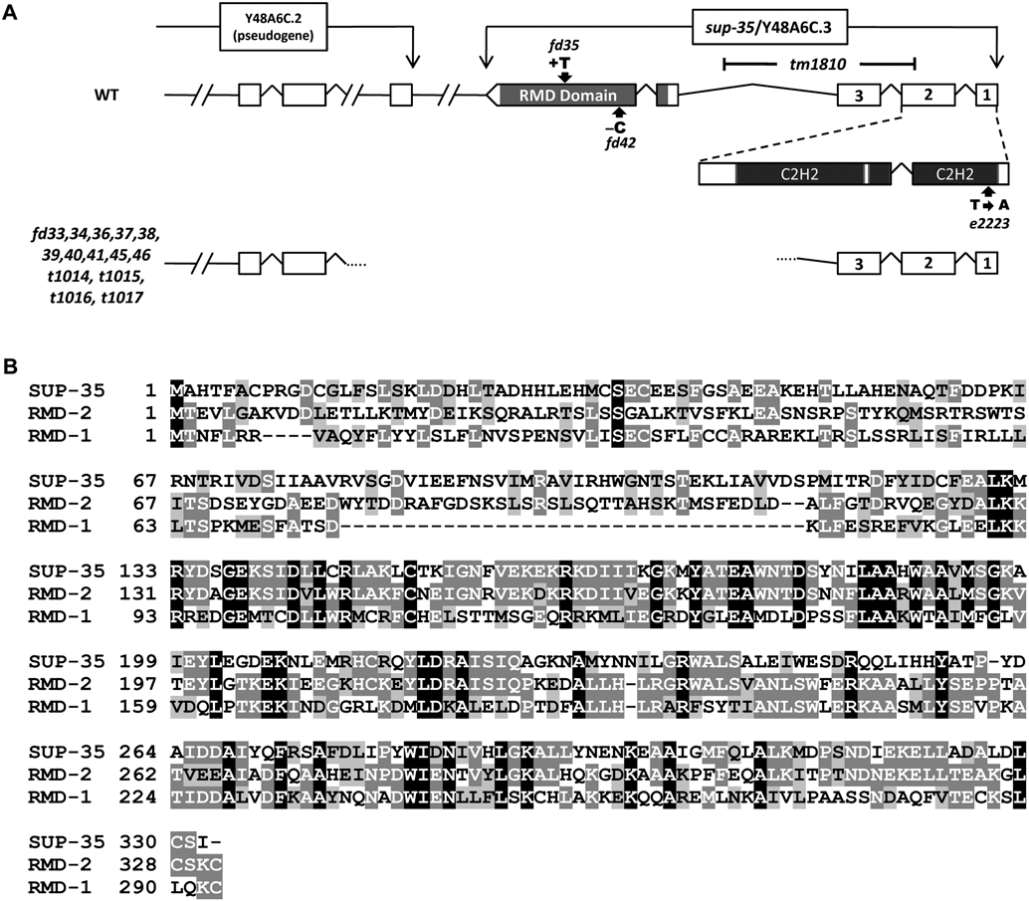
*sup-35* genomic locus. (A) Genomic organization of *sup-35* showing the locations of the two N-terminal C2H2-type Zn-finger domains and the C-terminal RMD-like domain. *sup-35* alleles that contain point mutations or single nucleotide insertions or deletions are indicated by bold arrows. The large deletion alleles are represented by a gap in the gene structure; dotted lines indicate the approximate position of the deletion breakpoints as determined by PCR. The consortium generated *tm1810* deletion allele is indicated by the black bracket. *tm1810* deletes 741 nucleotides and leads to translational termination following amino acid 62. (B) Peptide sequence alignment showing the similarity of SUP-35 to RMD-2 and RMD-1. Consensus amino acids are indicated by white letters with black backgrounds, identical residues by white letters with dark-gray backgrounds, and similar residues by black letters with light-gray backgrounds. Overall, SUP-35 is 37% identical to RMD-2 and 19% identical to RMD-1. RMD-2 and RMD-1 are 30% identical.

Additional support for Y48A6C.3 as the affected locus was provided by sequencing both Y48A6C.1 and Y48A6C.3 in *sup-35(e2223) pha-1(ts)* double mutants. We detected a T-to-A transversion at nucleotide position 19 of the Y48A6C.3 open reading frame, resulting in the conversion of a cysteine to a serine at amino acid position seven. In contrast, we failed to identify any differences in the Y48A6C.1 locus between the published wild-type (N2) and *sup-35(e2223)* mutant sequences. Furthermore, we identified sequence alterations in Y48A6C.3 in five previously isolated alleles of *sup-35* [31] as well as in 14 additional alleles identified by our laboratory. A summary of our sequence analysis is shown in Figure 1A. Two alleles, *fd35* and *fd42*, contained a single nucleotide insertion and deletion, respectively, leading to frameshifts within exon 5 of Y48A6C.3. All other *sup-35* alleles contained either large deletions or insertions within Y48A6C.3, and are presumed to be null alleles (Figure 1A and data not shown). Taken together, our findings strongly indicate that *sup-35* is encoded by Y48A6C.3. Furthermore, given that the majority of these alleles were identified as spontaneous revertants ([31] and this work), the *sup-35* genomic region would appear to be unusually unstable and subject to recombination events that lead to gross alterations of the locus.

Based on the WormBase predicted gene model, as well as an ORFeome-generated full-length cDNA, *sup-35* encodes a 332-amino-acid protein containing two N-terminal C2H2-type Zn-finger domains along with two tetratrico peptide repeats (TPR) at its C terminus. The molecular lesion identified in *sup-35(e2223)* is predicted to disrupt the first Zn finger, indicating that this domain is likely to be essential for SUP-35 function. The presence of the Zn-finger motifs suggests a potential role for SUP-35 in transcriptional regulation. Alternatively, the Zn-fingers may be involved in protein-RNA, protein-protein, or protein-lipid interactions.

Interestingly, other than its close paralog Y48A6C.1, SUP-35 is most similar to an evolutionarily conserved family of RMD (regulators of microtubule dynamics) proteins (Figure 1B; [32]. Of the six RMD family members in *C. elegans*, SUP-35 is most similar to RMD-2; the C-terminal 215 amino acids of SUP-35 are 52% identical to a corresponding region in RMD-2, which in turn shares greater homology with SUP-35 than with other *C. elegans* RMD proteins (Figure 1B and data not shown). Interestingly, RMD-2, along with RMD-1 and RMD-3, can physically associate with microtubles in vitro [32]. Consistent with the RMD-like domain of SUP-35 having an important functional role is the observation that two alleles of *sup-35*, *fd35* and *fd42*, may specifically affect this region of the protein. Nevertheless, SUP-35 differs from other *C. elegans* RMD family members, as well as RMD proteins in other organisms, by the presence of its unique N-terminal Zn-finger domains. The TPR domains in SUP-35 suggest a possible role in protein-protein interactions [33].

### SUP-35 shows a dynamic pattern of expression during embryogenesis

To assess the pattern of SUP-35 expression during development, multiple independent transgenic strains were generated expressing full-length SUP-35 fused to GFP under the control of the native *sup-35* promoter/enhancer region (also see Materials and Methods). For reasons described below, the SUP-35::GFP expression analysis was performed in *sup-36* and *sup-37* mutant backgrounds, both of which gave identical results.

**Figure 2:**
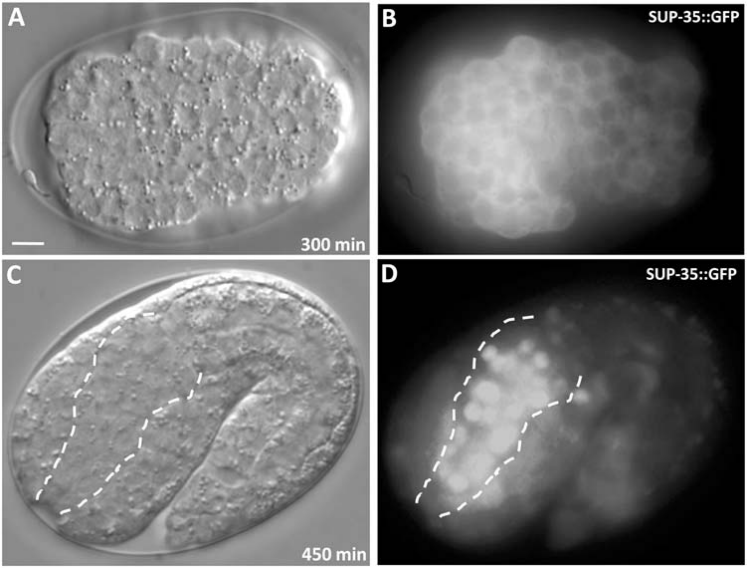
SUP-35::GFP expression. (A–D) DIC (A,C) and corresponding GFP (B,D) images of SUP-35::GFP (*fdEx57*) expression in embryos. The SUP-35::GFP reporter encodes a full-length functional fusion protein that is expressed under the control of the native *sup-35* promoter. Prior to overt morphogenesis (A,B), SUP-35::GFP expression is predominantly cytoplasmic. After the initiation of body morphogenesis, SUP-35::GFP becomes enriched in nuclei (C,D). The developing primordial pharynx is outlined by dashed lines in C and D. The embryos depicted are homozygous for a mutation in *sup-36(e2217)* identical results were obtained using an independently generated array (*fdEx58*). Identical results were also obtained using analogous arrays in the *sup-37(e2215)*background. Anterior is to the left. Scale bar in A, 10 µm for A–D.

SUP-35::GFP expression was first observed in embryos at around the 50- to 100-cell stage. Expression of SUP-35::GFP was ubiquitous throughout the proliferative phase of embryogenesis and was strongly enriched in the cytoplasm (Figure 2A and 2B). Commensurate with the onset of visible morphogenesis (∼400 minutes), SUP-35::GFP localization became pronounced in nuclei, most notably in cells comprising the pharyngeal primordium (Figure 2C and 2D). Pharyngeal cells also maintained nuclear SUP-35::GFP expression throughout larval stages and into adulthood (data not shown). In addition, weaker SUP-35::GFP could be detected in the nuclei of several non-pharyngeal cells in the posterior.

### Mutations in *sup-35* suppress synthetic pharyngeal defects

Mutations in either *sup-36* or *sup-37* are capable of suppressing all pair-wise combinations of mutations in *lin-35*, *ubc-18*, and *pha-1* [29]. Consistent with this, the same constellation of synthetic-lethal mutations was efficiently suppressed by loss of *sup-35* (Table 1). This includes suppression by the canonical allele of *sup-35*, *e2223*; a consortium-generated deletion allele, *tm1810*; and by *sup-35(RNAi)*. Suppression by *sup-35(tm1810)* also further confirms the molecular identity of this locus.

Previous studies from our laboratory have implicated the RING finger–domain protein, ARI-1, as the primary co-partner of UBC-18 in the regulation of pharyngeal development [34]. Consistent with this, a consortium-generated deletion allele of *ari-1*, *tm2549*, showed strong synthetic interactions with *pha-1(ts)*, and this lethality was suppressed by *sup-35(RNAi)* (Table 1). Taken together, these findings suggest that *sup-35* functions within a regulatory network that includes *pha-1*, *lin-35*, *ubc-18*, and *ari-1* to control pharyngeal development.

### *sup-35* suppression of *pha-1* mutations requires residual PHA-1 activity

Extragenic suppression in *C. elegans* arises through a number of distinct mechanisms [35]. Such mechanisms can, in some cases, be distinguished based on whether or not suppression occurs in the presence of a null allele. For this reason, we first sought to determine whether the strongest characterized allele of *pha-1*, *e2123ts*, retains activity at the non-permissive temperature of 25°C; *e2123ts* is a missense mutation that leads to a conversion of cysteine to tyrosine at amino acid position 169 of PHA-1 [36]. We thus generated high-copy extrachromosomal arrays carrying the *pha-1(ts)* variant in mutant animals that were already chromosomally homozygous for the *pha-1(ts)* mutation. We then assayed for the ability of *pha-1(ts)* high-copy overexpression to rescue the lethal phenotype of *pha-1(ts)* mutants at 25°C. If the protein product of *pha-1(ts)* were to retain residual activity at 25°C, we would expect to see some suppression of *pha-1(ts)* temperature sensitivity. As shown in Table 2, overexpression of *pha-1(ts)* efficiently rescued defects associated with genomic *pha-1(ts)* loss of function, indicating that, at 25°C, *pha-1(e2123ts)* does not behave as a null allele.

**Table 2:** *pha-1(e2123ts)* retains partial activity at non-permissive temperatures.

Genotype	Total Embryos	% Embryonic lethality at 25°C^a^	% Larval lethality at 25°C^a^
*pha-1(e2123ts)*	384	94.2	5.7
*pha-1(e2123ts); fdEx51*	128 (GFP+)	0	0
	199 (GFP−)	94.9	5.0
*pha-1(e2123ts); fdEx53*	141 (GFP+)	0	0
	255 (GFP−)	94.5	5.4

The independently derived extrachromosomal arrays *fdEx51* and *fdEx53* carry multiple copies of the *pha-1(e2123ts)* allele in addition to the *sur-5*::GFP marker.

a Both embryonic- and larval-lethal animals exhibited the Pun phenotype.

**Figure 3:**
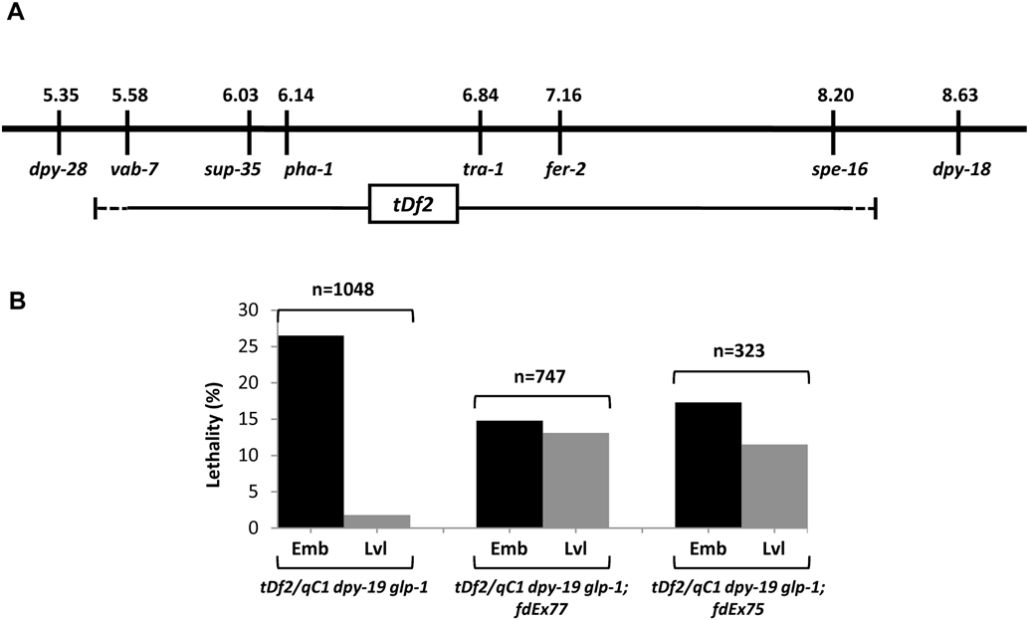
Analysis of a chromosomal deficiency that removes both *sup-35* and *pha-1*. (A) Genetic map showing extent of the *tDf2* deficiency. Although the precise molecular endpoints are unknown, *tDf2* minimally extends from *vab-7* to *spe-16* but does not encompass *dpy-28* and *dpy-18*. Based on an analysis of genes within the region, *tDf2* leads to the deletion of 48 to 74 genes, including *sup-35* and *pha-1*. (B) Percentage of embryonic- and larval-lethal F1 animals observed for each of the indicated parental genotypes. *tDf2/qC1 dpy-19 glp-1* is a balanced strain that segregates 25% *tDf2/tDf2* homozygous progeny. *fdEx77* and *fdEx75* are independently generated extrachromosomal arrays that express wild-type *pha-1* and the *sur-5*::GFP marker. Note that strains carrying the *pha-1*-rescuing arrays display a pronounced reduction in the frequency of embryonic lethality along with a concomitant increase in larval lethality. Furthermore, the decrease in embryonic lethality is proportional to the transmission frequencies of the individual arrays; *fdEx77* and *fdEx75* exhibit transmission frequencies of 50% (n = 140) and 39% (n = 182), respectively. In addition, for parental strains harboring either the *fdEx77* or *fdEx75* arrays, >80% of the observed larval-lethal F1 progeny expressed GFP. Emb, embryos; Lvl, L1 larvae.

Given the absence of a well-characterized null allele of *pha-1*, we decided to make use of a regional deficiency on chromosome III, *tDf2*, which removes both the *pha-1* and *sup-35* loci, as well as 46–72 additional genes (Figure 3A). Previous analysis, along with our current work, indicates that homozygous *tDf2*/*tDf2* mutants arrest as embryos that display a phenotype closely resembling *pha-1* strong loss-of-function mutations, suggesting that *pha-1* may be the earliest-acting zygotic gene within the region deleted by the deficiency [31]. If so, then the apparent lack of suppression observed in *tDf2*/*tDf2* embryos, where both *sup-35* and *pha-1* are deleted, would suggest that loss of *sup-35* cannot suppress the *pha-1* null phenotype. Alternatively, another early-acting gene within the deficiency, one that is not suppressed by loss of *sup-35*, could be responsible for the *pha-1*-like phenotype observed in *tDf2*/*tDf2* homozygotes.

To distinguish between these two possibilities, we introduced an extrachromosomal array containing wild-type copies of *pha-1* into a balanced strain that carries the *tDf2* deficiency (*tDf2/qC1 dpy-19 glp-1*). In the absence of any array, this strain segregates ∼25% *tDf2*/*tDf2* progeny that arrest as dead embryos with morphological defects similar to those observed for *pha-1(ts)* mutants at 25°C (Figure 3B). Strikingly, in the presence of *pha-1* rescuing arrays, we observed a substantial decrease in the frequency of embryonic lethality (Figure 3B). This effect was observed using multiple independently generated arrays, with the extent of embryonic rescue corresponding closely to the transmission frequencies of the individual arrays (Figure 3B and data not shown). Furthermore, we observed a proportional increase in the percentage of array-positive larval-lethal animals (Figure 3B), indicating that some other gene within the deficiency is required for progression through larval development. Taken together, these results demonstrate that *pha-1* is the earliest-acting zygotic gene within *tDf2* and, most importantly, that loss of *sup-35* cannot suppress the *pha-1* null genotype. These findings are also consistent with the observation that *sup-35 pha-1(e2123)/tDf2* animals, which carry only a single copy of the *pha-1* hypomorphic allele, display much weaker suppression than that of *sup-35 pha-1(e2123)* animals, which retain two copies of this allele (data not shown; [31].

As an additional test, we made use of two recently generated deletion alleles of *pha-1* (*tm3671* and *tm3569*; gift of National Bioresource Project). *tm3671* is a 203-bp deletion that removes part of the second exon of *pha-1*, creating a premature stop codon after 30 amino acids and is a presumed null allele. *tm3569* contains an in-frame 568-bp deletion extending from exon 2 through exon 4, which removes 149 amino acids of PHA-1 (isoform Y48A6C.5a). Both *pha-1(tm3671)/+* and *pha-1(tm3569)/+* heterozygous hermaphrodites produce ∼25% embryonic-lethal F1 progeny that phenocopy *pha-1(ts)* embryos (at 25°C). Consistent with our deficiency analysis, growth of *pha-1(tm3671)/+* and *pha-1(tm3569)/+* heterozygotes on *sup-35(RNAi)* failed to decrease the percentage of embryonic-arrested progeny, further indicating that reduction of *sup-35* activity cannot suppress complete loss of function of *pha-1* (data not shown). In contrast, *sup-35(RNAi)* efficiently suppressed the lethality of *pha-1(ts)* mutants (at 25°C), as well as all tested synthetic phenotypes (Table 1).

### SUP-35 is a transcriptional repressor of *pha-1*

**Figure 4:**
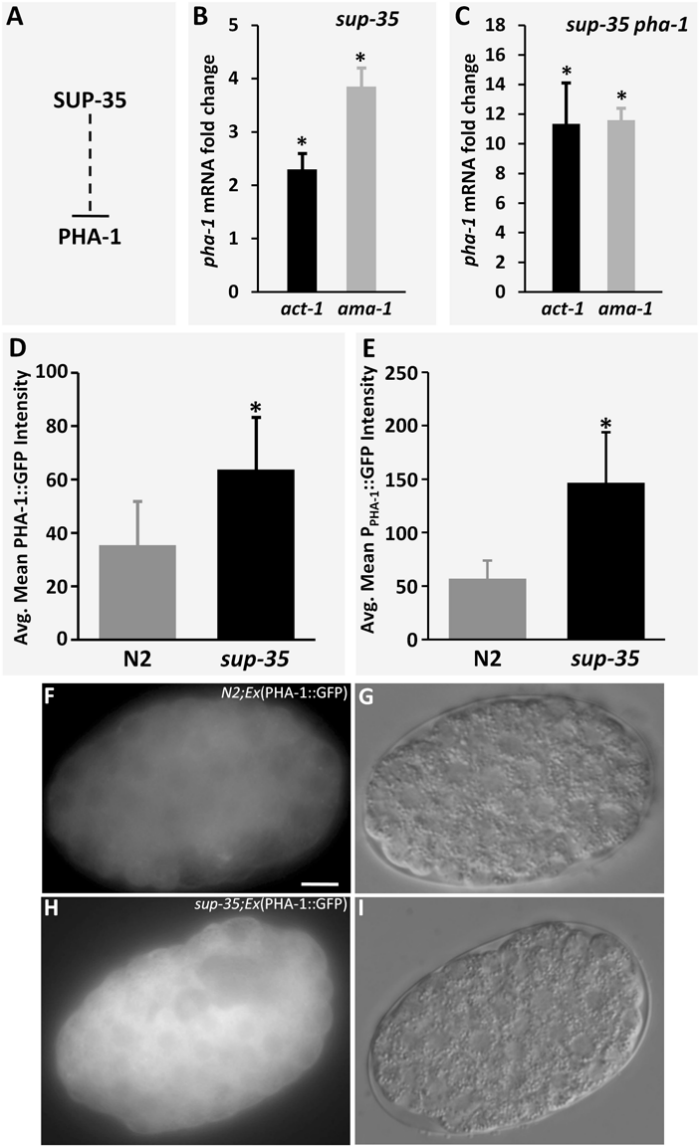
SUP-35 negatively regulates *pha-1*. (A) Testable model for the regulation of PHA-1 by SUP-35. (B,C) Quantification of endogenous *pha-1* mRNA levels in embryos by qRT-PCR in *sup-35(tm1810)* single mutants (B) or *sup-35(e2223) pha-1(e2123* double mutants (C) using *act-1* (black bar) and *ama-1* (gray bar) as loading controls. Fold changes were obtained after normalizing to wild type (B) or *pha-1(e2123)* single mutants (C). Error bars represent s.e.m. Means of the indicated groups were analyzed for significance using a two-tailed Student's *t*-test (*p<0.0). Quantification of PHA-1::GFP (D) and P*_pha-1_*::GFP (E) fluorescence in wild-type and *sup-35(tm1810)*mutants. The average mean GFP intensity for each genotype was analyzed for significance using a two-tailed Student's t-test (*p<0.0001). (F–I) Representative GFP (F,H) and corresponding DIC (G,I) images of PHA-1::GFP expression in wild type (F,G) and *sup-35(tm1810)*mutants (H,I). Digital camera exposure times were identical for all embryos assayed. Mean GFP intensities were determined as described in Materials and Methods. Scale bar in F, 10 µm for F–I.

Given that loss of *sup-35* cannot suppress the *pha-1* null genotype, we hypothesized that SUP-35 may function as a negative upstream regulator of *pha-1*. Furthermore, because SUP-35 contains C2H2-type Zn fingers that are critical for its activity (Figure 1A), we reasoned that SUP-35 may mediate repression of *pha-1* at the level of transcription (Figure 4A). Consistent with this, qRT-PCR experiments revealed embryonic *pha-1* mRNA levels to be 2- to 4-fold more abundant in *sup-35(tm1810)* mutants as compared with wild type using two independent internal normalization controls (Figure 4B). An even greater increase in *pha-1* mRNA levels was observed in embryos from *sup-35(e2223) pha-1(ts)* double mutants relative to *pha-1(ts)* single mutants (Figure 4C). This latter result is significant in that *pha-1* mRNA levels were assessed in a genetic background in which PHA-1 activity is compromised. The observed difference in the degree to which *pha-1* is upregulated in these strains could reflect a heightened sensitivity to SUP-35 levels in the *pha-1* mutant background or could be due to differences between the two *sup-35* alleles used in these studies.

As a second test, we made use of a previously described strain that expresses a functional full-length PHA-1::GFP fusion protein [29]. Because this fusion protein is regulated by sequences derived from the native *pha-1* promoter, its expression should be sensitive to alterations in the activities of endogenous transcriptional regulators. Consistent with data obtained from qRT-PCR, PHA-1::GFP was upregulated at least 2-fold in *sup-35(tm1810)* mutants relative to wild-type embryos (Figure 4D and 4F–4I; Figure S1). These findings also indicate that changes in *pha-1* mRNA levels lead to corresponding changes in the abundance of PHA-1 protein.

The above results indicate that SUP-35 may negatively regulate *pha-1* at the level of transcription or mRNA stability. To distinguish between these possibilities, we assayed expression levels of a P*_pha-1_*::GFP reporter [29] in wild-type and *sup-35* mutants. Because this construct contains only the 5′ upstream regulatory region of *pha-1*, effects on mRNA stability through the *pha-1* 3′UTR should not be observed. Using this reporter, we observed that P*_pha-1_*::GFP is upregulated ∼3-fold in *sup-35* mutants versus wild-type embryos (Figure 4E; Figure S1). Taken together, these data provide strong evidence that SUP-35 normally functions to inhibit *pha-1* at the level of transcription.

### SUP-35 acts genetically upstream of *sup-36* and *sup-37* to inhibit *pha-1*

If SUP-35 negatively regulates *pha-1*, then *sup-35* overexpression should cause a reduction in PHA-1 levels and therefore would be expected to phenocopy *pha-1* loss-of-function mutations. Consistent with this, extensive attempts to revert the suppression of *sup-35; pha-1* mutants through the expression of wild-type *sup-35* via an extrachromosomal array failed to generate stable transgenic lines. This includes experiments in which *sup-35* was engineered to be present at low copy numbers. In addition, *sup-35* transgenic expression was also highly toxic to wild-type animals, as was expression of the SUP-35::GFP fusion protein. Given that SUP-35 may require the *pha-1* suppressors SUP-36 and SUP-37 to mediate its activities, we hypothesized that SUP-35 overexpression may not be toxic in genetic backgrounds that remove either *sup-36* or *sup-37* activities. Consistent with this prediction, we encountered no difficulties in obtaining stable transgenic lines carrying wild-type *sup-35* (or SUP-35::GFP) at high copy number in either the *sup-36* or *sup-37* mutant background (also see Materials and Methods). This finding indicates that SUP-36 and SUP-37 function genetically downstream of SUP-35. However, SUP-36 and SUP-37 could conceivably function upstream of SUP-35 if they are required for SUP-35 activation.

**Figure 5:**
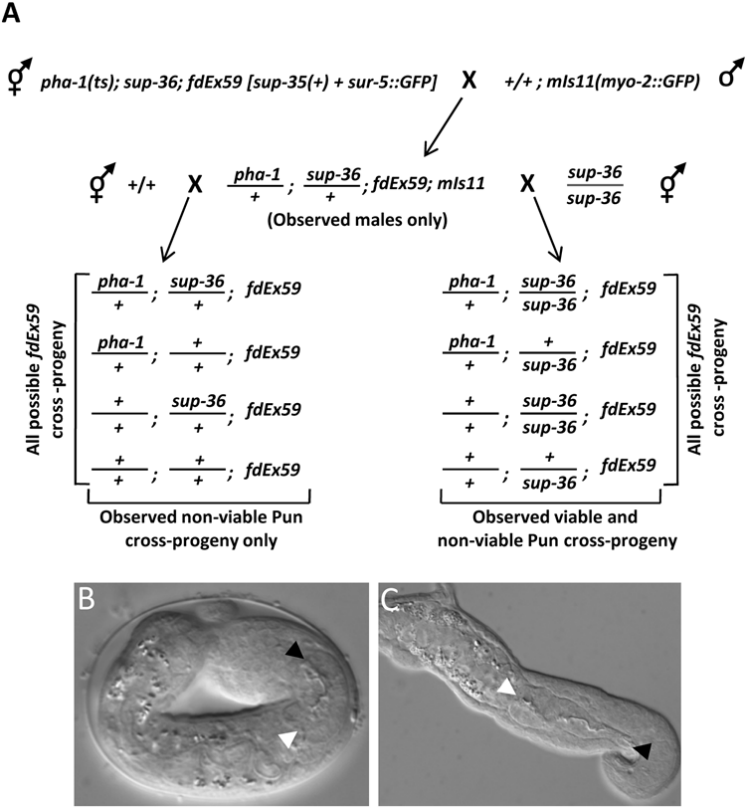
SUP-35 overexpression phenocopies *pha-1* loss of function. (A) Representative genetic strategy applied to assay the effects of SUP-35 overexpression in wild type and in *sup-36* and *sup-37* mutant backgrounds. Wild-type males carrying an integrated *myo-2*::GFP reporter (*mIs11*) were crossed into *pha-1*; *sup-36* hermaphrodites carrying a *sup-35*-overexpressing extrachromosomal array *(fdEx59)*. Although this mating failed to produce viable F1 cross-progeny hermaphrodites, fertile cross-progeny males were generated, which were identified by expression of the *myo-2*::GFP reporter. F1 cross-progeny males were then mated to wild-type or *sup-36* hermaphrodites, and cross-progeny were identified based on the *myo-2*::GFP reporter. Mating into the N2 strain failed to produce viable cross-progeny males or hermaphrodites, whereas mating to *sup-36* generated both viable and non-viable male and hermaphrodite F2 cross-progeny. Non-viable F2 cross-progeny from both matings displayed a Pun (Pharynx unattached) phenotype, and these animals uniformly carried the *fdEx59* array. Identical results were also obtained for *sup-37* mutants using the above strategy, and similar findings were obtained for both *sup-36* and *sup-37* using additional genetic approaches (see Materials and Methods). (B,C) DIC images of a typical non-viable embryo (B) and larva (C) obtained through the above mating. Note the Pun phenotype. Black and white arrowheads indicate the anterior and posterior pharyngeal boundaries, respectively. Scale bar in C, 1 µm for B,C.

To determine directly the phenotypic effects of SUP-35 overexpression in a wild-type background, we performed a series of genetic crosses, an example of which is shown in Figure 5A. *sup-35* overexpression was toxic to hermaphrodites that expressed both *sup-36* and *sup-37* zygotically, even if *sup-36* or *sup-37* were absent maternally. Surprisingly, *sup-35* overexpression was not toxic in males that expressed both *sup-36* and *sup-37* zygotically, provided that either *sup-36* or *sup-37* maternal contributions were absent. In contrast, *sup-35* overexpression was toxic to both males and hermaphrodites when both *sup-36* and *sup-37* were present maternally and zygotically. Most strikingly, non-viable *sup-35*-overexpressing embryos and larvae obtained through these crosses had a phenotype that was identical to strong *pha-1* loss-of-function mutations (Figure 5B and 5C). Taken together, these results are consistent with our finding that SUP-35 functions as a negative regulator of *pha-1* and further indicate that SUP-35 acts together with SUP-36 and SUP-37 to control *pha-1* expression levels.

### SUP-35 acts through *pha-1* to suppress synthetic pharyngeal defects

Our above analyses strongly indicate that *sup-35* suppression of partial loss-of-function mutations in *pha-1* occurs through the upregulation of *pha-1* mRNA, which in turn leads to increased PHA-1 protein levels (Figure 4). An extension of this model is that suppression of the synthetic pharyngeal genotypes by *sup-35* (Table 1) may occur through an identical mechanism. If that is the case, then an increase in PHA-1 levels, even in the presence of wild-type *sup-35*, should be sufficient to suppress the synthetic phenotype of *lin-35; ubc-18* double mutants. To address this, we overexpressed wild-type PHA-1 from high-copy extrachromosomal arrays in *lin-35; ubc-18* double mutants and assayed for rescue. Strong suppression of synthetic lethality was observed in three out of three independent transgenic lines, leading to the generation of viable double mutant strains that carried only the PHA-1-overexpression transgenic array. This finding is consistent with the hypothesis that *sup-35*-mediated suppression of *pha-1(ts)* and the synthetic genotypes occurs through the same mechanism.

A second prediction of the above model is that inhibition of *pha-1* activity should revert the suppression observed in *lin-35; sup-35(tm1810) ubc-18* triple mutants (Table 1). We therefore subjected triple mutants to *pha-1(RNAi)* feeding and assayed for loss of suppression. Whereas 100% (n = 255) of *lin-35; sup-35 ubc-18* animals reached adulthood when grown on vector-RNAi control plates, only 12.9% (n = 200) of triple mutants grown on *pha-1(RNAi)* escaped embryonic or early-larval arrest. This finding further supports the model that *sup-35*-mediated suppression of both strong loss-of-function *pha-1* mutants and the synthetic genotypes occurs through the common mechanism of increasing PHA-1 levels.

### LIN-35, UBC-18–ARI-1, and HCF-1 function upstream of SUP-35 to regulate PHA-1 expression

**Figure 6:**
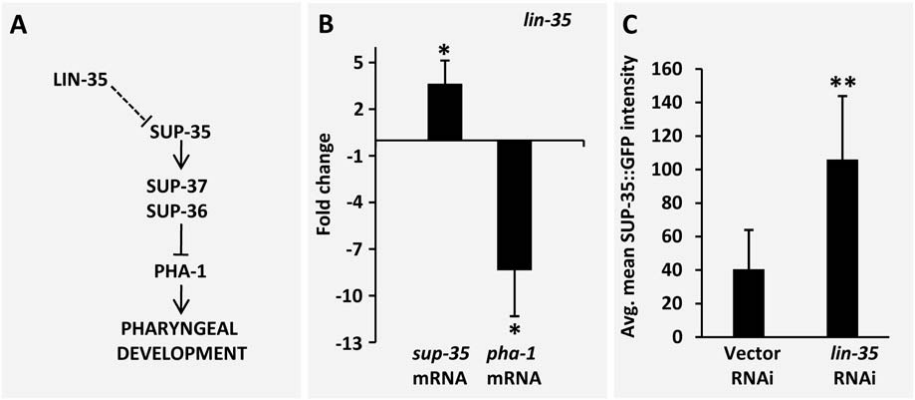
Regulation of *sup-35* and *pha-1* by LIN-35. (A) Testable model for the regulation of SUP-35 and PHA-1 by LIN-35. (B) Quantification of endogenous *sup-35* and *pha-1* mRNA levels in embryos by qRT-PCR in *lin-35* mutants using *act-1* as an internal normalization control. Fold changes were obtained after normalizing to N2. Note that consistent with *sup-35* mRNA upregulation in *lin-35* mutants, *pha-1* mRNA is significantly downregulated. (C) Quantification of SUP-35::GFP protein levels in embryos following RNAi inhibition of *lin-35* (also see Figure S1). Animals assayed were of genotype *pha-1(e2123); sup-36(e2217); fdEx57* (SUP-35::*GFP*) embryos of the genotype *pha-1(e2123); sup-37(e2215); fdEx63* (SUP-35::*GFP* showed similar trends (data not shown). Error bars represent s.e.m. The means of the indicated groups were analyzed for significance using a two-tailed Student's t-test. *p<0.05 and **p<0.0001.

**Figure 7:**
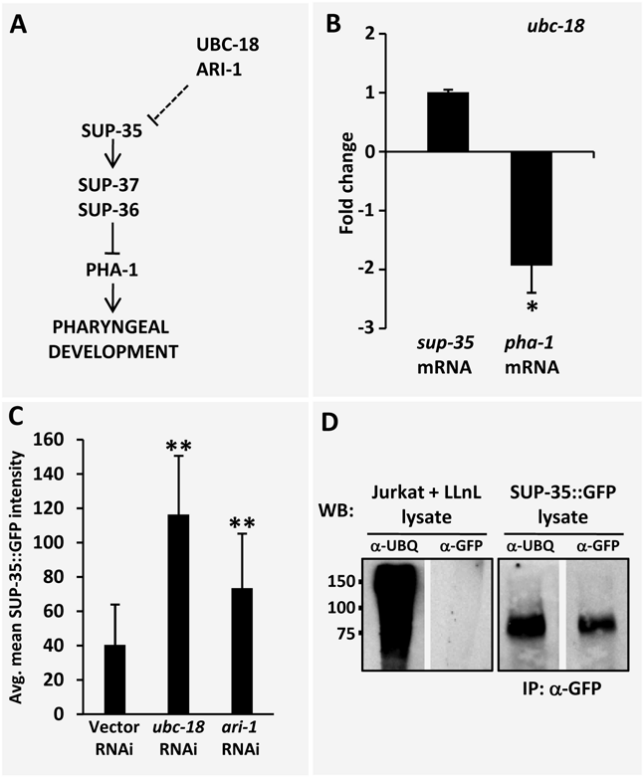
Regulation of *sup-35*and *pha-1* by UBC-18–ARI-1. (A) Testable model for the regulation of SUP-35 and PHA-1 by UBC-18–ARI-1. (B) Quantification of *sup-35* and *pha-1* endogenous mRNA levels in embryos by qRT-PCR in *ubc-18(ku354* mutants using *act-1* as an internal normalization control. Fold changes were obtained after normalizing to N2. Note that although *pha-1* mRNA was significantly decreased in *ubc-18* mutants, *sup-35* mRNA levels were unaffected. (C) Quantification of SUP-35::GFP protein levels in embryos following RNAi inhibition of *ubc-18* and *ari-1* (also see Figure S1). Note that SUP-35::GFP protein levels were substantially increased following inhibition of *ubc-18* and *ari-1*. Animals assayed were of genotype *pha-1(e2123); sup-36(e2217); fdEx57* (SUP-35::*GFP*) embryos of the genotype *pha-1(e2123); sup-37(e2215); fdEx63* (SUP-35::*GFP* showed similar trends (data not shown). Error bars (B and C) represent s.e.m. The means of the indicated groups were analyzed for significance using a two-tailed Student's t-test (*p<0.05 and **p<0.000). (D) SUP-35::GFP was immunoprecipitated from whole worm lysates of strain WY518 using polyclonal anti-GFP antibodies. The immunoprecipitates were analyzed by western blot using monoclonal antibodies to either GFP or ubiquitin. Lysates from Jurkat cells grown in the presence of the proteosomal inhibitor LLnL were used as a positive control for ubiquitinated products and as a negative control for GFP. The expected size of the non-ubiquitinated SUP-35::GFP fusion protein is ∼68 kDa.

In considering potential regulatory networks that could account for both the molecular and genetic data described above, we were able to construct a relatively straightforward model. In this scenario, LIN-35, functioning as a transcriptional repressor (Figure 6A), and UBC-18–ARI-1, acting as a complex to promote target protein degradation (Figure 7A), are negative regulators of SUP-35. Thus in *lin-35; ubc-18* double mutants, increased levels of SUP-35 would lead to the inhibition of PHA-1 and associated defects in pharyngeal development.

We first tested this model by examining the role of LIN-35 in the expression of endogenous *sup-35*. Consistent with the model, embryonic levels of *sup-35* mRNA are increased ∼4-fold in *lin-35* mutants as compared with wild type (Figure 6B). Correspondingly, SUP-35::GFP was upregulated 2- to 3-fold in embryos following *lin-35(RNAi)* treatment (Figure 6C; Figure S2), indicating that changes in *sup-35* mRNA levels are further reflected by changes in the abundance of SUP-35 protein. Most importantly, we observed an ∼5- to 10-fold reduction in the levels of endogenous *pha-1* mRNA in embryos derived from *lin-35* mutants versus those from wild type (Figure 6B). This latter result also provides an explanation for why mutations in *lin-35* are strongly synthetic with hypomorphic mutations in *pha-1* (also see Discussion).

We next examined the roles of UBC-18 and ARI-1 in the regulation of SUP-35 and PHA-1. In contrast to findings from *lin-35* mutants, embryonic *sup-35* mRNA levels in *ubc-18* mutants were identical to those observed in wild type (Figure 7B). Nonetheless, embryonic SUP-35::GFP protein levels were substantially increased following RNAi inhibition of *ubc-18* or *ari-1* (Figure 7C; Figure S2). These results indicate that UBC-18–ARI-1 negatively regulates SUP-35 post-transcriptionally, possibly at the level of SUP-35 stability. Consistent with this, we find that SUP-35::GFP is a target for ubiquitination in cell extracts from whole worms (Figure 7D). Furthermore, we observed that the increase in SUP-35 levels in *ubc-18* mutants correlates with a decrease in the expression levels of *pha-1* mRNA (Figure 7B). These findings, in combination with other molecular and genetic data, strongly support the model that LIN-35 and UBC-18–ARI-1 promote *pha-1* transcription by inhibiting SUP-35 expression and stability.

In previous studies, we have implicated the *C. elegans* E2F ortholog, EFL-1, as a regulatory partner of LIN-35 in the control of pharyngeal development [29], and have also defined the *C. elegans* E2F consensus binding motif [13]. Consistent with a role for E2F in the regulation of *sup-35*, we identified three candidate E2F bindings sites within the first 700 bp of the *sup-35* promoter region. One of these sites, located approximately 230 bp upstream of the predicted transcriptional start site (GATTCGCGCCT), conformed to all published criteria, suggesting that E2F may potentially regulate *sup-35* directly.

**Figure 8:**
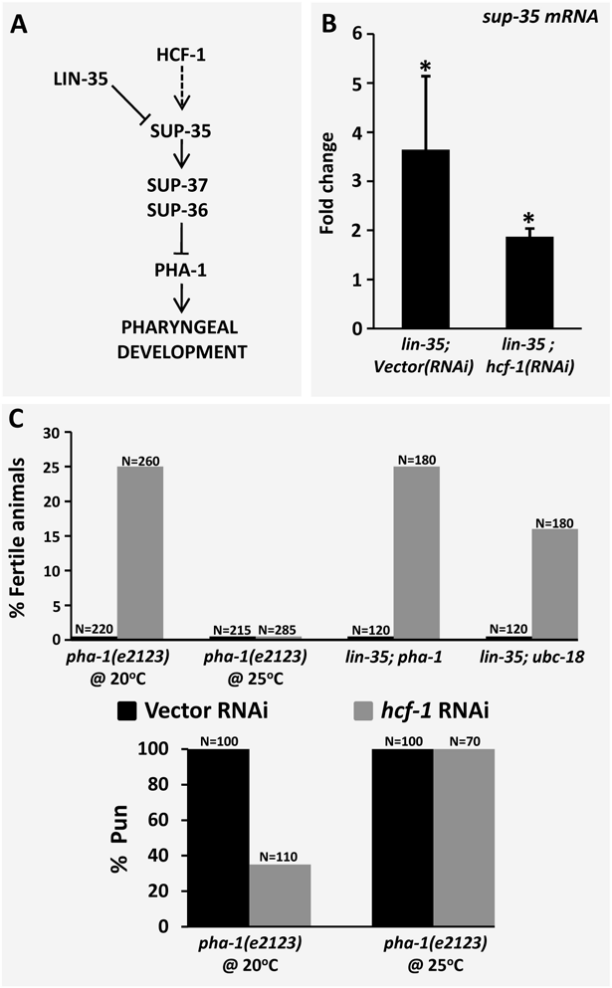
Regulation of *sup-35* by HCF-1. (A) Testable model for the regulation of *sup-35* by *hcf-1*. (B) Quantification of endogenous *sup-35* mRNA levels by qRT-PCR in *lin-35* and *lin-35; hcf-1(RNAi)* embryos using *act-1* as an internal normalization control. Note that *sup-35* mRNA levels drop ∼2 fold in *hcf-1*(RNAi)-treated *lin-35* mutants as compared with vector RNAi-treated *lin-35* single mutants. Fold changes were obtained after normalizing to N2. Error bars represent s.e.m and significance was calculated using a two-tailed Student's t-test (*p<0.05). (C) Suppression of larval arrest in *pha-1(ts)* and synthetic mutants by *hcf-1*(RNAi). *hcf-1(RNAi)* effectively increases the frequency of fertile adults by suppressing the larval lethality of *lin-35;pha-1* and *lin-35;ubc-18* double mutants. Similar results were also observed for *pha-1(ts)* at the intermediate temperature of 20°C, but not at 25°C. (D) *hcf-1*(RNAi) strongly suppresses the Pun (Pharynx unattached) in *pha-1(ts)* mutants at 20°C, but not 25°C. These results were also duplicated using RNAi injection to target a different region of the *hcf-1* transcript (Materials and Methods and data not shown).

Studies in mammals have implicated HCF-1 (host cell factor 1), as an important physical and functional co-partner of E2F in the activation of E2F target genes [37],[38]. For example, loss of HCF-1 activity in hamster cells leads to a reduction in the expression of E2F-regulated genes required for G_1_ entry resulting in arrest in G_0_ [39]. Interestingly, this G_0_ arrest can be bypassed through the inhibition of pRb family members, indicating that mammalian HCF-1 and pRb carry out opposing functions on E2F targets [40]. The presence of a structurally and functionally conserved ortholog of HCF-1 in *C. elegans* [41]–[43], led us to hypothesize that a similar regulatory relationship may exist in *C. elegans* (Figure 8A). To test this, we assayed levels of *sup-35* mRNA in *lin-35* mutants subjected to *hcf-1(RNAi)* by qRT-PCR. Notably, we observed an ∼2-fold reduction in the levels of *sup-35* mRNA in *lin-35; hcf-1(RNAi)* embryos as compared with *lin-35* mutants treated with a control RNAi (Figure 8B).

To see if the observed reduction in *sup-35* mRNA levels by *hcf-1(RNAi)* has a functional consequence in *lin-35; ubc-18* and *lin-35; pha-1* double mutants, we carried out *hcf-1(RNAi)* in these backgrounds and assayed for suppression of larval arrest, leading to the generation of fertile adults. Notably, reduction of *hcf-1* activity led to pronounced suppression of arrest in both *lin-35; ubc-18* and *lin-35; pha-1* mutant backgrounds (Figure 8C). We note that the partial phenotypic suppression of the synthetic mutants by *hcf-1(RNAi)* is consistent with the incomplete correction of *sup-35* overexpression in *lin-35; hcf-1(RNAi)* embryos (Figure 8B). In addition, *hcf-1(RNAi)* resulted in suppression of *pha-1(ts)* mutants at the intermediate temperature of 20°C, leading to a marked decrease in L1-larval pharyngeal defects and a corresponding increase in the frequency of fertile adults (Figure 8C).

## Discussion

### Identification and characterization of SUP-35

We report here the molecular identification and analysis of SUP-35. We provide evidence that loss of *sup-35* activity specifically suppresses the embryonic- and larval-lethal phenotypes of *pha-1* hypomorphic alleles. Additionally, loss of *sup-35* activity efficiently suppressed the synthetic lethal phenotypes of *lin-35; pha-1* and *lin-35; ubc-18* double mutants, as well as a number of related genotypes. *sup-35* is predicted to encode a C2H2-type Zn-finger protein, consistent with a role in transcriptional regulation (Figure 1), although other functional activities associated with Zn fingers domains are possible. Based on sequence similarity, SUP-35 is also a new member of the RMD family of proteins, several of which have been shown to associate with microtubules [32]. During early embryonic development, a functional SUP-35::GFP protein was expressed predominantly in the cytoplasm of most or all cells. Notably, at the onset of morphogenesis, SUP-35::GFP expression became enriched in pharyngeal nuclei (Figure 2). How this dynamic pattern of expression, as well as a potential association with microtubules, may contribute to the functions and regulation of SUP-35 is currently unclear. Further studies of SUP-35, as well as the additional suppressors SUP-36 and SUP-37, should shed light on these facets of SUP-35 regulation.

### A model for the redundant regulation of PHA-1

**Figure 9:**
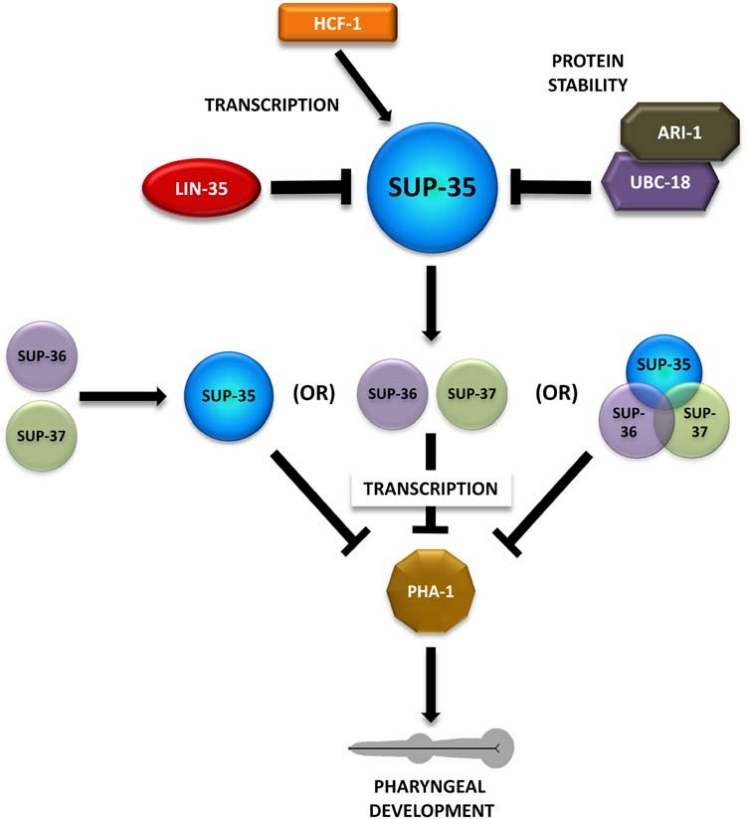
Model for the regulation of PHA-1. Schematic of the regulatory circuit acting upstream of PHA-1. We note that regulation of SUP-35 by LIN-35, HCF-1, and UBC-18–ARI-1 may not be direct, but could involve intermediate steps. For additional details, see text.

In previous work, we have shown that LIN-35, a transcriptional repressor, and UBC-18–ARI-1, an E2-E3 ubiquitin ligase complex, redundantly regulate pharyngeal morphogenesis [21],[34]. In addition, mutations in *lin-35*, *ubc-18*, and *ari-1* strongly enhance the pharyngeal morphogenetic defects of partial loss-of-function mutations in *pha-1* [29],[34]. In our current study, we provide both molecular and genetic evidence that LIN-35 and UBC-18–ARI-1 function as negative regulators of SUP-35, which in turn functions as a transcriptional repressor of *pha-1*. Thus, in our model, both LIN-35 and UBC-18–ARI-1 are positive, albeit indirect, regulators of PHA-1 through the inhibition of SUP-35 (Figure 9).

Evidence to support this model includes the findings that *pha-1* overexpression efficiently rescued the synthetic lethality of *lin-35; ubc-18* double mutants and that the suppression observed in *lin-35; ubc-18 sup-35* triple mutants was reversed by *pha-1(RNAi)*. Furthermore, *sup-35* overexpression in a wild-type background phenocopied *pha-1* loss of function (Figure 5). Consistent with the genetic data, qRT-PCR and GFP reporters indicate that *sup-35* mRNA and protein levels were upregulated in embryos where *lin-35* activity had been compromised, whereas *ubc-18* and *ari-1* specifically affected SUP-35 protein levels (Figure 6). Additionally, endogenous *pha-1* mRNA levels were decreased in *lin-35* and *ubc-18* mutants, whereas *pha-1* mRNA and protein levels were increased in *sup-35* mutants (Figure 6, Figure 7). This model accounts for both the synthetic lethality of *lin-35; ubc-18* double mutants as well as the genetic interactions observed between *pha-1* and *lin-35*, *ubc-18*, and *ari-1*, as *pha-1* hypomorphic mutations would be expected to be hypersensitive to conditions that further reduce *pha-1* mRNA levels.

An additional prediction of this model is that strong loss-of-function *pha-1* mutants should minimally phenocopy the defects observed in *lin-35; ubc-18* and *lin-35; pha-1* mutants. Specifically, *lin-35; ubc-18* and *lin-35; pha-1* mutants show early-stage defects in the re-orientation of anterior epithelial cells within the pharyngeal primordium [21],[29]. Surprisingly, however, we had previously failed to observe re-orientation defects in *pha-1(ts)* embryos grown at 25°C [29], even though these mutants show severe pharyngeal morphogenesis defects at later stages [29],[30]. We have subsequently repeated these experiments and, consistent with our earlier study, find little or no evidence for early-stage morphogenesis defects in *pha-1(ts)* embryos grown at the non-permissive temperature on either NGM or vector-RNAi control plates (data not shown). In contrast, *pha-1(ts)* mutants grown at 16°C on *pha-1(RNAi)* plates did display early-stage pharyngeal morphogenesis defects, demonstrating that a specific reduction in *pha-1* activity can phenocopy the early-stage defects observed in the synthetic mutants (data not shown). Moreover, the frequency and severity of *pha-1(ts); pha-1(RNAi)* morphogenesis defects were similar to those observed for *pha-1(ts)*; *lin-35(RNAi)* and *pha-1(ts); ubc-18(RNAi)* embryos grown at 16°C (data not shown). These observations indicate that early-stage defects in *pha-1(ts)* mutants are suppressed by growth at 25°C, suggesting an effect of temperature on the underlying process of cell re-orientation. Most importantly, these findings are internally consistent with our model, in which PHA-1 levels are positively regulated by LIN-35 and UBC-18 through the inhibition of SUP-35 (Figure 9).

Our observation that mutations in *sup-36* and *sup-37* abolish SUP-35-mediated toxicity indicate that *sup-36* and *sup-37* act genetically downstream of SUP-35. Thus, SUP-36 and SUP-37 may potentially function downstream of SUP-35 in a linear pathway to control *pha-1* expression. Alternatively, SUP-36 and SUP-37 may act in a complex with SUP-35, or in a parallel pathway that is required for SUP-35 activation (Figure 9).

We also find that inhibition of *hcf-1* by RNAi leads to a partial, though significant, suppression of larval arrest in *lin-35; ubc-18* and *lin-35; pha-1* mutants as well as the substantive suppression of both the L1 arrest and Pun (Pharynx unattached) phenotypes of *pha-1(ts)* mutants at 20°C. This genetic suppression correlates well with the observed decrease in *sup-35* mRNA levels in *lin-35; hcf-1(RNAi)* embryos. These results are consistent with our current model as well as previously published findings on mammalian HCF-1 [29],[44], and append our model with the addition of a phylogenetically-conserved component of the E2F network (Figure 9). Our finding also indicates that additional novel suppressors may be identified through the use of sensitized strains.

Elucidating the mechanistic bases of synthetic genetic interactions will continue to be a major challenge for the field of developmental genetics. These types of interactions will also likely be critical to our understanding of complex disease traits in humans. For example, a recent commentary in the New England Journal of Medicine states that “many, rather than few, variant risk alleles are responsible for the majority of the inherited risk of each common disease” [45].

Our current analysis provides a straightforward model to account for the genetic redundancies observed in an additional case study. Although understanding different sets of genetic interactions will undoubtedly require unique solutions, we contend that certain patterns of redundancy are likely to emerge. In this case, we have shown that a redundancy between a transcriptional regulator, LIN-35, and a mediator of protein stability, UBC-18–ARI-1 can be explained through the negative regulation of a common target, SUP-35. Similarly, we have previously shown that LIN-35 and FZR-1, a substrate-specificity component of the APC (anaphase-promoting complex) E3 ligase, mutually inhibit the expression levels of G1 cyclins [14]. Thus, a potential theme to emerge from our studies is the redundant control of common targets through distinct mechanisms of negative regulation. Additional studies into synthetic phenotypes in *C. elegans* and other systems should further elucidate general themes that may govern genetic redundancy.

## Materials and Methods

### Strains and maintenance

*C. elegans* were maintained using standard procedures [46]. Strains used in our analysis include GE24 [*pha-1(e2123)*], GE348 [*dpy-18 sup-35(e2223) pha-1(e2123)*], WY83 [*lin-35; ubc-18; kuEx119(lin-35+; sur-5::GFP*], WY119 [*lin-35; pha-1(fd1); kuEx119*], *sup-35*(*tm1810*), WY477 [*dpy-18 pha-1(e2123)*; *ari-1(tm2549)*], WY482 [*sup-35*(*tm1810*); SM469 (*PHA-1*::GFP; pRF4 *rol-6gf*)], WY527–528, [*lin-35;ubc-18; kuEx119; fdEx72–73 (pBX;rol-6(su1006gf))*], WY529–530 [*lin-35; ubc-18; fdEx72–73*] GE2158 [*tDf2/qC1 dpy-19(e1259) glp-1(q339)*], WY539–542 [*unc-13 lin-35; dpy-17 ubc-18 sup-35(tm1810)*], GE348 [*dpy-18 sup-35(e2223) pha-1(e2123ts)*], GE551 [*vab-7(e1562) sup-35(t1013) pha-1(e2123ts)*], GE552 [*vab-7(e1562) sup-35(t1014) pha-1(e2123ts)*], GE913 [*vab-7(e1562) sup-35(t1016) pha-1(e2123ts)*], GE914 [*vab-7(e1562) sup-35(t1015) pha-1(e2123ts)*], GE915 [*vab-7(e1562) sup-35(t1017) pha-1(e2123ts)*], and WY453–466 [*sup-35 (fd33–46) pha-1(e2123ts)*]. SM35 [PHA-1::GFP], SM36 [P*_pha-1_*::GFP].

To analyze SUP-35 overexpression and toxicity, the following strains were generated using either a *sup-35* genomic fragment or a cloned *sup-35*:GFP construct: WY512–513 [*pha-1(e2123ts); sup-36(e2217); fdEx57–58 (sup-35::GFP; rol-6)*], WY514–517 [*pha-1(e2123ts); sup-36(e2217); fdEx59–62 (sup-35 genomic fragment; sur-5::GFP)*], WY518 [*pha-1(e2123ts); sup-37(e2215); fdEx63 (sup-35::GFP; rol-6)*], WY519–520 [*pha-1(e2123ts); sup-37(e2215); fdEx64–65(sup-35 genomic fragment; sur-5::GFP)*], WY523–524 [*dpy-11 sup-3; fdEx68–69 (sup-35 genomic fragment;sur-5::GFP)*]; WY525–526, [*dpy-11 sup-3; fdEx70–71 (sup-35::GFP; rol-6)*].

Strains used for rescue analysis of pha-1(e2123ts) and the chromosomal deficiency tdf2 included WY506–511 [*pha-1(e2123ts); fdEx51–56(pBX/e2123; sur-5::GFP)*] and WY531–534 [*tDf2/qC1 dpy-19(e1259) glp-1(q339); fdEx74–77*(pBX; *sur-5::GFP)*].

*lin-35(n745; ubc-18(ku354 sup-35(tm1810)* triple mutants were generated by crossing *sup-35(tm1810)/+* males to *dpy-17 ubc-18 unc-32* hermaphrodites. Cross-progeny were allowed to self, and the resulting Dpy non-Unc recombinants were assayed for the *sup-35*(*tm1810*) deletion by PCR. Confirmed *dpy-17 ubc-18 sup-35(tm1810)* triple-mutant hermaphrodites were then crossed to *unc-13 lin-35/+* males. Following selfing of the cross-progeny, Dpy Unc animals were confirmed for *lin-35(n745)*, *ubc-18 (ku354)*, and *sup-35*(*tm1810*) by PCR and DNA sequencing.

To test for rescue of *lin-35; ubc-18* double mutants by *pha-1* overexpression, plasmid pBX, which contains a rescuing segment of the *pha-1* genomic locus [47], was co-injected with pRF4, which contains the dominant *rol-6(su1106)* marker [48], into strain WY83. Stable double transgenics were recognized by the presence of rolling *sur-5::GFP(+)* animals. Rescue was then determined by the presence of rolling viable non-GFP adults that could be further propagated in the absence of *kuEx119*.

### Construction of plasmids

A SUP-35::GFP fusion (pDF101)was constructed as follows. An ∼2.5-kb *sup-35* genomic fragment, which includes the upstream *sup-35* promoter/enhancer region, was amplified using the primer pair 5′-GCTCTAGATGATAGTCGTGTCGGTGGTCGTC-3′ and 5′-CGCGGATCCAATTGAGCACAAGTCAAGGGCGTCG-3′. This fragment was digested with *Bam*HI and *Xba*I and cloned in-frame into a similarly restricted pPD95.77 vector (gift of A. Fire). All recombinant clones were verified by restriction digestion and sequencing.

For the rescue of *pha-1(e2123ts)* mutants by *pha-1(e2123ts)* overexpression, a fragment of the *pha-1* genomic locus was amplified from *pha-1(e2123ts)* mutants using the primer pair 5′-CAGGACAATGATCTCGCCTT-3′ and 5′-TATCTTTTCACATGGAATACATGTAG3′ and digested with *Sal*I and *Bsa*BI. This fragment was then used to replace the analogous region of pBX. Recombinant plasmids carrying the *e2123ts* mutation were identified by digestion with *Bst*1107I, which recognizes the SNP created by the *e2123ts* point mutation, and further confirmed by sequencing.

### RNAi

RNAi feeding was carried out using standard protocols, and plates were cultured at 25°C to score for suppression [49]. The RNAi constructs *JA:Y48A6C.3*, *JA:Y48A6C.5*, and *JA:R01H12.6* were used to target *sup-35*, *pha-1*, and *ubc-18* gene products, respectively. RNAi constructs used to target *lin-35* and *ari-1* were previously described [14],[34]. *hcf-1(RNAi)* feeding was carried out using construct *JA:C46A5.9*, corresponding to exons 2–4. RNAi injection of *hcf-1* was carried out by gonadal injection of dsRNA (∼1.0 mg/ml) corresponding to exons 5 and 6.

### Fluorescence microscopy and measurements

Fluorescence microscopy was performed using a Nikon Eclipse microscope. Quantification of the GFP fluorescence in embryos was carried out using Open Lab Software Version 5.0.2. All images were captured using identical exposure times, and all embryos used in our analysis were of similar developmental stages (∼200–300 cells). An average of the mean fluorescence was calculated to compare GFP expression levels. P values were determined using a Student's t-test.

### SUP-35 overexpression and toxicity

Because multicopy transgene expression of SUP-35 and SUP-35::GFP was toxic in wild-type backgrounds, arrays were initially generated in *sup-36* and *sup-37* mutants. To determine the effect of SUP-35 and SUP-35::GFP overexpression in wild-type animals, males of genotype +/+; *mIs11 (myo-2::GFP)* were crossed to *pha-1(e2123ts)*; *sup-36*; *fdEx59* hermaphrodites. *fdEx59* expresses wild-type *sup-35* and the co-injection marker *sur-5::GFP*. Such crosses resulted in the generation of *fdEx59+* F1 males only, which were identified by the presence of both *sur-5::GFP* and *myo-2::GFP*. F1 males were then mated to either N2 hermaphrodites or homozygous *sup-36* hermaphrodites. When the F1 males were crossed to *sup-36* hermaphrodites, non-viable Pun and viable cross-progeny animals were obtained, whereas all the cross-progeny from the N2 hermaphrodite matings were non-viable and exhibited the Pun phenotype. These results were reproduced using three independently generated extrachromosomal arrays in both *sup-36* and *sup-37* mutant backgrounds. Similar results were also obtained for the SUP-35::GFP construct co-injected with pRF4.

As an alternative approach, males of the genotype *dyp-13 unc-24/+* were crossed to *pha-1(e2123ts); sup-36; fdEx59* hermaphrodites. F1 hermaphrodites were placed on individual plates and allowed to self; cross-progeny were determined by the presence of Dpy Unc animals. In the event that SUP-35 overexpression was non-toxic, half of the cross-progeny F1s [*pha-1(e2123ts)/+;sup-36/dpy-13 unc-24; fdEx59*] should have segregated one-sixteenth of the F2 animals with a genotype of *+/+; dpy-13 unc-24; fdEx59*. Although our crosses resulted in a high frequency of F1 cross-progeny males, they failed to produce F1 hermaphrodites that segregated Dpy Unc F2 animals. To extend these results, F1 cross-progeny males were subsequently crossed to N2 hermaphrodites. This cross resulted in *fdEx59+* animals that arrested uniformly as arrested embryos or larvae that exhibited the Pun phenotype. Again, these results were reproducible with other independently generated arrays and when analogous crosses were performed in the *sup-37* mutant background

### qRT–PCR

Strains were grown at 16°C and total RNA from bleached embryos was isolated using the Trizol reagent (Invitrogen) followed by phenol-chloroform extraction. All samples were DNase (Invitrogen ) treated and cleaned using the RNeasy Midi Kit (Qiagen). cDNA was synthesized using random primers and Superscript reverse transcriptase II (Invitrogen) at 42°C for 1 hour. First-strand cDNA was purified using the Qiagen Microelute Kit and eluted in 10 l final volume.

Primer pairs used for the various genes include *pha-1* [5′-TCGACTGGAGCTTCGTGTAAGTCA-3′ and 5′-ACGGTGCAAGGGCATTAAGGAAAC-3′]; *ama-1* [5′-TGATGTGATGACTGCGAAGGGACA-3′ and 5′-TTCGAATGAACAACGCATCAGGGC-3′]; *act-1* [5′-TTACTCTTTCACCACCACCGCTGA-3′ and 5′-TCGTTTCCGACGGTGATGACTTGT-3′]; and *sup-35* [5′-GATCATGCGAGCGGTTATTCGTC-3′ and 5′-GATCGATGGACTTCTCTCCAGAA-3′]. All primer pairs amplified regions that spanned sizeable introns such that cDNA amplification was strongly favored. Furthermore, we did not detect genomic contamination in our cDNA samples based on several tests including gel-purified amplimer band sizes. Primer pairs used for the *act-1* internal normalization are predicted to amplify *act-1–3*. Primer pairs used for the *ama-1* were specific to this gene. qRT-PCR was performed using a BioRad icycler in a total reaction volume of 50 l using the BioRad SYBR green supermixwith the following reaction conditions: initial denaturation at 95°C for 3 min, followed by 40 cycles of denaturation at 95°C for 30 seconds and a combined annealing and extension step at 60°C for 30 seconds. After the final amplification cycle, a melt curve analysis was performed to examine the specificity of the reaction. The fold-change of the mRNA levels was calculated by the delta-delta Ct method For each qRT-PCR experiment, amplification was done in triplicate for both the test and the normalization genes, and the results were checked for reproducibility using at least one biological duplicate. In addition, all data were reproduced using at least two biological replicates. P values were determined using a Student's t-test.

### Immunoprecipitation and western blotting

Mixed-stage worms from 10 large NGM-OP50 plates were pooled and washed with M9 and distilled water and resuspended in 500 µl of homogenization buffer (20 mM Tris-HCl pH 7.5, 100 mM NaCl, 5 mM MgCl_2_, 1 mM EGTA, 1 mM DTT, 1% TritonX-100, protease inhibitors). Worms were then sonicated, incubated on ice, and lysates were cleared of large particles by centrifugation. To immunoprecipitate SUP-35::GFP, precleared worm lysate was incubated with 5 µg of polyclonal anti-GFP antibody (Santa Cruz) at 4°C for 2 hrs and the resulting immune complex was pulled down using 30 µl of proteinA-sepharose beads (Invitrogen) by over-night incubation at 4°C. Beads were washed 3× with cold homogenization buffer and subjected to SDS-PAGE and western blot analysis. Westerns to detect ubiquitinated products were carried out using either 2 µg of monoclonal anti-ubiquitin antibody (Santa Cruz) or 2 µg of monoclonal anti-GFP primary antibody (Invitrogen). Visualization was carried out using HRP-conjugated goat anti-mouse secondary antibodies (Santa Cruz) at 1∶5000 and peroxidase activity was detected by the enhanced chemiluminesence assay (Pierce). LLnL-treated Jurkat cell lysate (Santa Cruz) was used as a positive control for ubiquitination.

## Supporting Information

Figure S1 Quantification of PHA-1::GFP (A and B) and P*_pha-1_*::GFP (C and D) fluorescence intensities in individual embryos in N2 (A and C) and *sup-35*(*tm1810*) mutant backgrounds (B and D).
(0.20 MB TIF)

Figure S2 Quantification of SUP-35::GFP fluorescence intensities in individual embryos following treatment of strains with vector RNAi (A), *lin-35(RNAi)* (B), *ubc-18(RNAi)* (C), and *ari-1(RNAi)* (D).
(0.23 MB TIF)

## References

[1] Fraser AG, Kamath RS, Zipperlen P, Martinez-Campos M, Sohrmann M (2000). Functional genomic analysis of C. elegans chromosome I by systematic RNA interference.. Nature.

[2] Kamath RS, Fraser AG, Dong Y, Poulin G, Durbin R (2003). Systematic functional analysis of the Caenorhabditis elegans genome using RNAi.. Nature.

[3] Simmer F, Moorman C, van der Linden AM, Kuijk E, van den Berghe PV (2003). Genome-wide RNAi of C. elegans using the hypersensitive rrf-3 strain reveals novel gene functions.. PLoS Biol.

[4] Fay DS, Yochem J (2007). The SynMuv genes of Caenorhabditis elegans in vulval development and beyond.. Dev Biol.

[5] Ferguson EL, Horvitz HR (1989). The multivulva phenotype of certain Caenorhabditis elegans mutants results from defects in two functionally redundant pathways.. Genetics.

[6] Ceol CJ, Horvitz HR (2004). A new class of C. elegans synMuv genes implicates a Tip60/NuA4-like HAT complex as a negative regulator of Ras signaling.. Dev Cell.

[7] Cui M, Chen J, Myers TR, Hwang BJ, Sternberg PW (2006). SynMuv genes redundantly inhibit lin-3/EGF expression to prevent inappropriate vulval induction in C. elegans.. Dev Cell.

[8] Sternberg PW (2005).

[9] van den Heuvel S, Dyson NJ (2008). Conserved functions of the pRB and E2F families.. Nat Rev Mol Cell Biol.

[10] Ceol CJ, Horvitz HR (2001). dpl-1 DP and efl-1 E2F act with lin-35 Rb to antagonize Ras signaling in C. elegans vulval development.. Mol Cell.

[11] Harrison MM, Ceol CJ, Lu X, Horvitz HR (2006). Some C. elegans class B synthetic multivulva proteins encode a conserved LIN-35 Rb-containing complex distinct from a NuRD-like complex.. Proc Natl Acad Sci U S A.

[12] Lu X, Horvitz HR (1998). lin-35 and lin-53, two genes that antagonize a C. elegans Ras pathway, encode proteins similar to Rb and its binding protein RbAp48.. Cell.

[13] Kirienko NV, Fay DS (2007). Transcriptome profiling of the C. elegans Rb ortholog reveals diverse developmental roles.. Dev Biol.

[14] Fay DS, Keenan S, Han M (2002). fzr-1 and lin-35/Rb function redundantly to control cell proliferation in C. elegans as revealed by a nonbiased synthetic screen.. Genes Dev.

[15] Boxem M, van den Heuvel S (2001). lin-35 Rb and cki-1 Cip/Kip cooperate in developmental regulation of G1 progression in C. elegans.. Development.

[16] Bender AM, Wells O, Fay DS (2004). lin-35/Rb and xnp-1/ATR-X function redundantly to control somatic gonad development in C. elegans.. Dev Biol.

[17] Cui M, Fay DS, Han M (2004). lin-35/Rb cooperates with the SWI/SNF complex to control Caenorhabditis elegans larval development.. Genetics.

[18] Cardoso C, Couillault C, Mignon-Ravix C, Millet A, Ewbank JJ (2005). XNP-1/ATR-X acts with RB, HP1 and the NuRD complex during larval development in C. elegans.. Dev Biol.

[19] Chesney MA, Kidd AR, Kimble J (2006). gon-14 functions with class B and class C synthetic multivulva genes to control larval growth in Caenorhabditis elegans.. Genetics.

[20] Bender AM, Kirienko NV, Olson SK, Esko JD, Fay DS (2007). lin-35/Rb and the CoREST ortholog spr-1 coordinately regulate vulval morphogenesis and gonad development in C. elegans.. Dev Biol.

[21] Fay DS, Large E, Han M, Darland M (2003). lin-35/Rb and ubc-18, an E2 ubiquitin-conjugating enzyme, function redundantly to control pharyngeal morphogenesis in C. elegans.. Development.

[22] Kirienko NV, McEnerney JD, Fay DS (2008). Coordinated regulation of intestinal functions in C. elegans by LIN-35/Rb and SLR-2.. PLoS Genet.

[23] Hsieh J, Liu J, Kostas SA, Chang C, Sternberg PW (1999). The RING finger/B-box factor TAM-1 and a retinoblastoma-like protein LIN-35 modulate context-dependent gene silencing in Caenorhabditis elegans.. Genes Dev.

[24] Lehner B, Calixto A, Crombie C, Tischler J, Fortunato A (2006). Loss of LIN-35, the Caenorhabditis elegans ortholog of the tumor suppressor p105Rb, results in enhanced RNA interference.. Genome Biol.

[25] Wang D, Kennedy S, Conte D, Kim JK, Gabel HW (2005). Somatic misexpression of germline P granules and enhanced RNA interference in retinoblastoma pathway mutants.. Nature.

[26] Schertel C, Conradt B (2007). C. elegans orthologs of components of the RB tumor suppressor complex have distinct pro-apoptotic functions.. Development.

[27] Grote P, Conradt B (2006). The PLZF-like protein TRA-4 cooperates with the Gli-like transcription factor TRA-1 to promote female development in C. elegans.. Dev Cell.

[28] Voutev R, Killian DJ, Ahn JH, Hubbard EJ (2006). Alterations in ribosome biogenesis cause specific defects in C. elegans hermaphrodite gonadogenesis.. Dev Biol.

[29] Fay DS, Qiu X, Large E, Smith CP, Mango S (2004). The coordinate regulation of pharyngeal development in C. elegans by lin-35/Rb, pha-1, and ubc-18.. Dev Biol.

[30] Schnabel H, Schnabel R (1990). An Organ-Specific Differentiation Gene, pha-1, from Caenorhabditis elegans.. Science.

[31] Schnabel H, Bauer G, Schnabel R (1991). Suppressors of the organ-specific differentiation gene pha-1 of Caenorhabditis elegans.. Genetics.

[32] Oishi K, Okano H, Sawa H (2007). RMD-1, a novel microtubule-associated protein, functions in chromosome segregation in Caenorhabditis elegans.. J Cell Biol.

[33] Das AK, Cohen PW, Barford D (1998). The structure of the tetratricopeptide repeats of protein phosphatase 5: implications for TPR-mediated protein-protein interactions.. Embo J.

[34] Qiu X, Fay DS (2006). ARI-1, an RBR family ubiquitin-ligase, functions with UBC-18 to regulate pharyngeal development in C. elegans.. Dev Biol.

[35] Hodgkin J (2005). Genetic suppression.. WormBook.

[36] Granato M, Schnabel H, Schnabel R (1994). Genesis of an organ: molecular analysis of the pha-1 gene.. Development.

[37] Knez J, Piluso D, Bilan P, Capone JP (2006). Host cell factor-1 and E2F4 interact via multiple determinants in each protein.. Mol Cell Biochem.

[38] Tyagi S, Chabes AL, Wysocka J, Herr W (2007). E2F activation of S phase promoters via association with HCF-1 and the MLL family of histone H3K4 methyltransferases.. Mol Cell.

[39] Goto H, Motomura S, Wilson AC, Freiman RN, Nakabeppu Y (1997). A single-point mutation in HCF causes temperature-sensitive cell-cycle arrest and disrupts VP16 function.. Genes Dev.

[40] Reilly PT, Wysocka J, Herr W (2002). Inactivation of the retinoblastoma protein family can bypass the HCF-1 defect in tsBN67 cell proliferation and cytokinesis.. Mol Cell Biol.

[41] Juang BT, Izeta A, O'Hare P, Luisi BF (2005). Purification and characterization of the Caenorhabditis elegans HCF protein and domains of human HCF.. Biochemistry.

[42] Lee S, Horn V, Julien E, Liu Y, Wysocka J (2007). Epigenetic regulation of histone H3 serine 10 phosphorylation status by HCF-1 proteins in C. elegans and mammalian cells.. PLoS ONE.

[43] Wysocka J, Liu Y, Kobayashi R, Herr W (2001). Developmental and cell-cycle regulation of Caenorhabditis elegans HCF phosphorylation.. Biochemistry.

[44] Cui M, Kim EB, Han M (2006). Diverse chromatin remodeling genes antagonize the Rb-involved SynMuv pathways in C. elegans.. PLoS Genet.

[45] Kraft P, Hunter DJ (2009). Genetic Risk Prediction — Are We There Yet?. N Engl J Med.

[46] Stiernagle T (2005).

[47] Granato M, Schnabel H, Schnabel R (1994). pha-1, a selectable marker for gene transfer in C. elegans.. Nucleic Acids Res.

[48] Mello CC, Kramer JM, Stinchcomb D, Ambros V (1991). Efficient gene transfer in C.elegans: extrachromosomal maintenance and integration of transforming sequences.. Embo J.

[49] Ahringer J (2005).

