# Supplementary figures and images for: A Mechanistic Basis for the Coordinated Regulation of Pharyngeal Morphogenesis in Caenorhabditis elegans by LIN-35/Rb and UBC-18–ARI-1
Source: PLoS Genet. 2009 Jun 12;5(6):e1000510. doi: 10.1371/journal.pgen.1000510 (PMC2686152; doi:10.1371/journal.pgen.1000510)

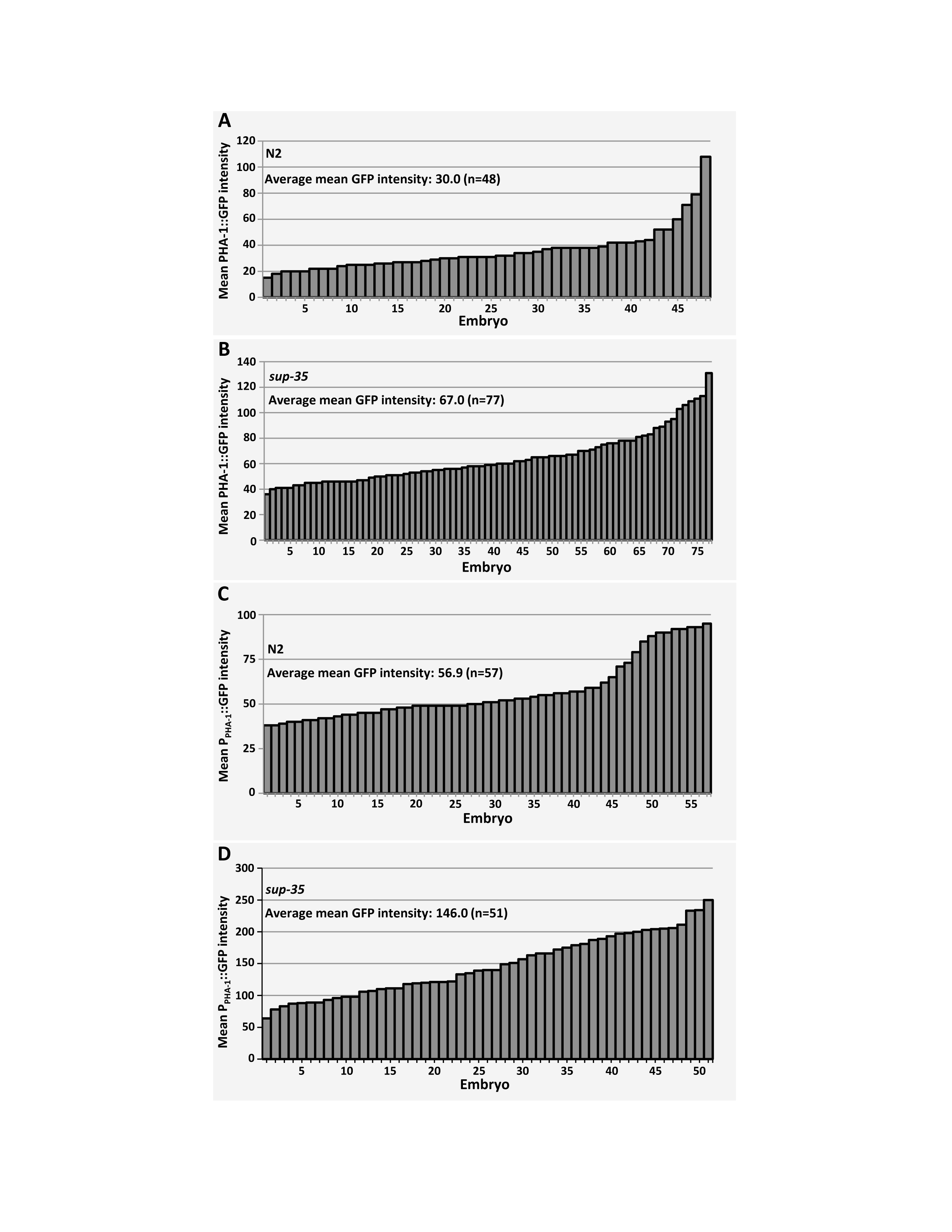

Supplement: Figure S1 — Quantification of PHA-1::GFP (A and B) and Ppha-1::GFP (C and D) fluorescence intensities in individual embryos in N2 (A and C) and sup-35(tm1810) mutant backgrounds (B and D). (0.20 MB TIF) [file pgen.1000510.s001.tif]

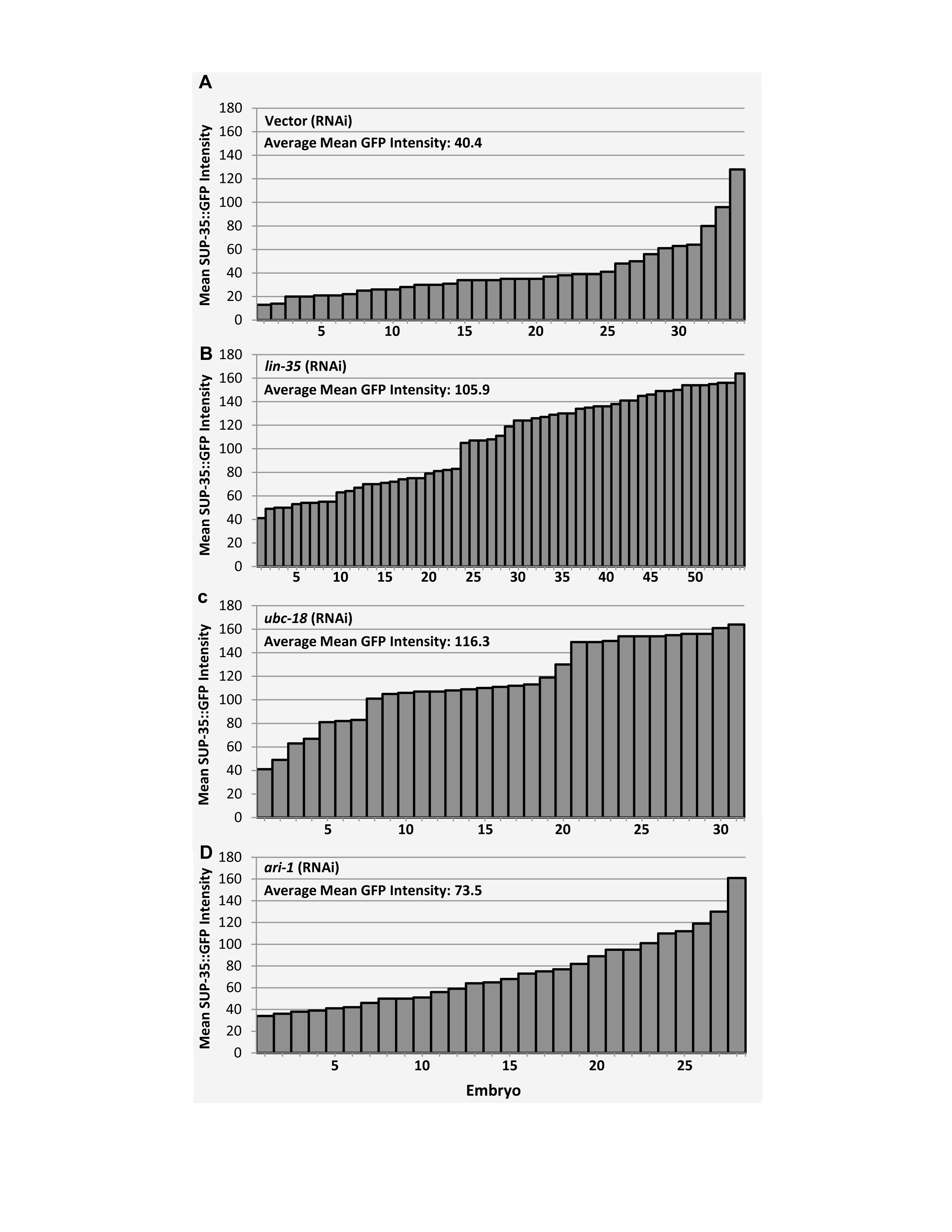

Supplement: Figure S2 — Quantification of SUP-35::GFP fluorescence intensities in individual embryos following treatment of strains with vector RNAi (A), lin-35(RNAi) (B), ubc-18(RNAi) (C), and ari-1(RNAi) (D). (0.23 MB TIF) [file pgen.1000510.s002.tif]
